# Impact of pulmonary emphysema on exercise capacity and its physiological determinants in chronic obstructive pulmonary disease

**DOI:** 10.1038/s41598-018-34014-5

**Published:** 2018-10-24

**Authors:** Benjamin M. Smith, Dennis Jensen, Marc Brosseau, Andrea Benedetti, Harvey O. Coxson, Jean Bourbeau

**Affiliations:** 1 McGill University Health Centre Research Institute, Montreal, Canada; 2 Columbia University Medical Center, New York, USA; 3 Department of Kinesiology and Physical Education, McGill University, Montreal, Canada; 4 Department of Epidemiology, Biostatistics and Occupational Health, McGill University, Montreal, Canada; 5 Department of Radiology, University of British, Columbia, Canada

## Abstract

Exercise limitation is common in chronic obstructive pulmonary disease (COPD). We determined the impact of pulmonary emphysema on the physiological response to exercise *independent* of contemporary measures of COPD severity. Smokers 40–79 years old with COPD underwent computed tomography, pulmonary function tesing, and symptom-limited incremental exercise testing. COPD severity was quantified according to the Global Initiative for Chronic Obstructive Lung Disease (GOLD) by spirometry (GOLD 1–4); and symptom burden and exacerbation risk (GOLD A-D). Emphysema severity was quantified as the percent lung volume <−950 Hounsfield units. Regression models adjusted for age, gender, body size, smoking status, airflow limitation, symptom burden and exacerbation risk. Among 67 COPD subjects (age 67 ± 8 years; 75% male; GOLD 1–4: 11%, 43%, 30%, 16%), median percent emphysema was 11%, and peak power output (PPO) was 61 ± 32 W. Higher percent emphysema independently predicted lower PPO (−24 W per 10% increment in emphysema; 95%CI −41 to −7 W). Throughout exercise, higher percent emphysema predicted 1) higher minute ventilation, ventilatory equivalent for CO_2_, and heart rate; and 2) lower oxy-hemoglobin saturation, and end-tidal PCO_2_. Independent of contemporary measures of COPD severity, the extent of pulmonary emphysema predicts lower exercise capacity, ventilatory inefficiency, impaired gas-exchange and increased heart rate response to exercise.

## Introduction

Chronic obstructive pulmonary disease (COPD) is a heterogeneous disorder characterized by persistent airflow limitation due to airway and alveolar abnormalities^1^. Exercise intolerance is observed at all severities of COPD, but correlates poorly with spirometry and responds variably to pharmacological interventions targeting airway bronchoconstriction and inflammation^2–7^. There is increasing interest in identifying COPD endotypes to better target the heterogeneous pathophysiologies that contribute to COPD^8^. Indeed, contemporary guidelines emphasize indivudalized patient management^1^.

Pulmonary emphysema is defined anatomically as enlargement of alveoli with destruction of their walls, and is present to varying degrees in COPD^9^. Emphysema is reliably quantified *in vivo* by computed tomography (CT) and correlates with histopathlogy^10,11^.

Emphysema at CT has been shown to predict mortality independent of airflow limitation^12,13^, suggesting a distinct pathophysiology from non-emphysematous COPD. Emphysema is also associated with impaired pulmonary blood flow, and cardiac filling that is independent of airflow limitation^14–17^. While emphysema has been shown to predict shorter six-minute walk distance, lower peak O_2_ uptake and greater exercise ventilatory inefficiency in COPD^18–29^, the independent contribution of emphysema in COPD to the abnormal physiological and perceptual response to exercise remains poorly understood.

We hypothesized that emphysema severity in patients with mild-to-very-severe COPD would be associated with lower exercise capacity and altered cardiac, metabolic, gas exchange, ventilatory and perceptual responses to symptom-limited incremental cycle exercise testing, independent of airflow limitation, symptom burden and exacerbation risk.

## Results

Among 70 participants enrolled in the study, 67 completed study measures and were included in the analysis. The mean age of participants completing the study was 67 ± 8 years, and 75% were men. The prevalence of GOLD 1–4 was 11%, 43%, 30%, and 16%, respectively; and GOLD group A-D was 49%, 25%, 8%, and 18%, respectively. Participant characteristics stratified by quartiles of percent emphysema are summarized in Table 1. Airflow limitation severity, gas-trapping, hyperinflation, and D_LCO_ impairment increased with percent emphysema quartile, whereas age, gender, height, BMI, pectoral muscle area, pulmonary artery-to-aorta diameter, and smoking history were similar.

**Table 1:** Participant characteristics by quartile of percent emphysema.

	Quartiles of Percent Emphysema				p-value
	Q1 N = 17	Q2 N = 17	Q3 N = 17	Q4 N = 16	
Percent emphysema	3.1 (2.1, 4.5)	8.4 (7.4, 9.6)	14.5 (12.6, 16.2)	27.5 (23.8, 35.5)	
Age – years	66 ± 8	65 ± 9	67 ± 8	70 ± 6	0.205
Male – %	65	76	76	81	0.151
Height – cm	167 ± 11	168 ± 11	167 ± 8	171 ± 7	0.311
Body mass index – kg/m^2^	26 ± 4	28 ± 7	28 ± 6	25 ± 5	0.404
Smoking status – %					0.004
Current	53	41	24	12	
Former	47	59	76	88	
Pack-years of smoking	59 ± 22	61 ± 32	56 ± 30	48 ± 26	0.154
mMRC dyspnea rating – %					0.171
0	41	29	24	13	
1	18	35	47	19	
2	29	6	6	25	
3	6	18	12	38	
4	6	12	12	6	
FEV_1_% predicted	68 ± 20	54 ± 18	52 ± 20	35 ± 16	<0.001
FVC % predicted	94 ± 20	89 ± 20	92 ± 23	81 ± 23	0.160
FEV_1_/FVC	0.54 ± 0.11	0.45 ± 0.12	0.41 ± 0.09	0.31 ± 0.08	<0.001
GOLD by severity of airflow limitation – %					<0.001
1	24	6	12	6	
2	59	59	47	6	
3	18	29	35	38	
4	0	6	6	50	
GOLD by group – %					0.056
A	53	59	59	25	
B	41	18	18	25	
C	6	6	12	6	
D	0	18	12	44	
Frequent or severe exacerbator – %	6	24	24	47	0.006
Residual volume % predicted	101 ± 58	131 ± 64	151 ± 55	175 ± 89	0.004
Functional residual capacity % predicted	141 ± 32	145 ± 43	157 ± 25	172 ± 38	0.012
Total lung capacity % predicted	113 ± 18	115 ± 18	122 ± 12	129 ± 14	0.005
CT lung volume/Plethysmographic TLC	0.70 ± 0.09	0.77 ± 0.08	0.73 ± 0.07	0.79 ± 0.11	<0.001
D_LCO_ % predicted	73 ± 14	58 ± 13	57 ± 19	31 ± 11	< 0.001
Pectoralis muscle area – cm^2^	34.5 ± 10.5	28.2 ± 8.1	31.0 ± 8.6	28.2 ± 7.6	0.090
Pulmonary artery to aorta diameter ratio	0.79 ± 0.12	0.76 ± 0.09	0.75 ± 0.11	0.82 ± 0.15	0.530
**Resting**					
${\dot{{\rm{V}}}O}_{2}$ – mL/kg/min	4.5 ± 1.5	4.8 ± 1.0	4.2 ± 1.2	4.4 ± 1.5	0.823
Dyspnea – Borg 0–10 scale	0.0 (0.0, 0.0)	0.0 (0.0, 1.5)	0.0 (0.0, 0.0)	1.0 (0.5, 3.0)	0.019
Leg fatigue – Borg 0–10 scale	0.0 (0.0, 0.0)	0.0 (0.0, 1.0)	0.0 (0.0, 0.0)	0.5 (0.0, 1.0)	0.112
${\dot{{\rm{V}}}}_{{\rm{E}}}$ – L/min	15.1 ± 3.5	17.3 ± 3.4	15.4 ± 3.0	16.4 ± 3.0	0.354
V_T_ − L	0.8 ± 0.1	0.9 ± 0.2	0.8 ± 0.2	0.8 ± 0.2	0.323
Respiratory rate – breaths/min	20 ± 4	21 ± 5	19 ± 3	21 ± 5	0.953
${\dot{{\rm{V}}}}_{{\rm{E}}}$/${\dot{{\rm{V}}}\text{CO}}_{2}$	57.8 ± 9.8	59.1 ± 13.5	57.4 ± 11.7	66.0 ± 12.6	0.086
P_ET_CO_2_ – mmHg	30.6 ± 3.4	29.7 ± 3.4	30.5 ± 5.3	27.7 ± 4.1	0.088
S_p_O_2_ – %	96.2 ± 3.1	96.0 ± 1.5	94.9 ± 1.4	95.7 ± 2.5	0.498
Heart rate – beats/min	82 ± 15	80 ± 14	84 ± 11	88 ± 14	0.052
O_2_ pulse – mL O_2_/beat	4.0 ± 1.3	4.8 ± 1.4	3.8 ± 0.7	3.5 ± 0.8	0.081
**Nadir** ${\dot{{\rm{V}}}}_{{\rm{E}}}$ **/** ${\dot{{\rm{V}}}\text{CO}}_{2}$					
${\dot{{\rm{V}}}}_{{\rm{E}}}$/${\dot{{\rm{V}}}\text{CO}}_{2}$	40.2 ± 5.3	46.0 ± 8.2	43.7 ± 9.7	50.2 ± 12.2	0.013
${\dot{{\rm{V}}}}_{{\rm{E}}}$ – L/min	38.6 ± 12.7	37.4 ± 14.7	38.9 ± 10.4	28.1 ± 7.8	0.015
${\dot{{\rm{V}}}\text{CO}}_{2}$ – L/min	1.0 ± 0.3	0.9 ± 0.4	0.9 ± 0.2	0.6 ± 0.1	<0.001
P_ET_CO_2_ – mmHg	36.2 ± 4.3	33.9 ± 4.5	36.4 ± 7.8	32.7 ± 6.3	0.230
${\dot{{\rm{V}}}O}_{2}$ – mL/kg/min	13.2 ± 4.8	11.6 ± 3.8	10.9 ± 3.1	8.8 ± 2.6	<0.001
Power output - W	50 ± 27	53 ± 21	57 ± 23	33 ± 17	0.002
**Peak Exercise**					
Power output - W	75 ± 38	65 ± 34	66 ± 26	38 ± 19	0.001
${\dot{{\rm{V}}}O}_{2}$ – mL/kg/min	14.7 ± 5.6	13.2 ± 5.8	12.2 ± 4.3	9.3 ± 3.0	<0.001
Dyspnea – Borg 0–10 scale	3.0 (3.0, 5.0)	3.0 (3.0, 4.0)	5.0 (3.0, 5.0)	5.0 (3.0, 5.0)	0.329
Leg fatigue – Borg 0–10 scale	4.0 (3.0, 4.0)	3.0 (3.0, 5.0)	4.0 (3.0, 4.0)	4.0 (2.0, 5.0)	0.668
${\dot{{\rm{V}}}}_{{\rm{E}}}$ – L/min	46.7 ± 18.3	47.0 ± 26.0	41.3 ± 14.4	31.5 ± 12.8	0.007
V_T_ – L	1.5 ± 0.5	1.5 ± 0.5	1.5 ± 0.5	1.4 ± 0.4	0.380
Respiratory rate – breaths/min	30 ± 5	32 ± 8	28 ± 4	24 ± 8	0.001
${\dot{{\rm{V}}}}_{{\rm{E}}}$/${\dot{{\rm{V}}}\text{CO}}_{2}$	43.7 ± 5.9	48.0 ± 8.7	45.8 ± 10.5	52.2 ± 11.8	0.034
P_ET_CO_2_ – mmHg	34.2 ± 4.0	32.9 ± 4.9	35.3 ± 7.0	31.8 ± 6.5	0.399
S_p_O_2_ – %	95.8 ± 1.9	94.9 ± 2.9	93.3 ± 4.1	93.4 ± 4.2	0.083
Heart rate – beats/min	118 ± 18	107 ± 18	117 ± 20	108 ± 15	0.454
O_2_ pulse – mL O_2_/beat	8.9 ± 3.2	9.5 ± 3.7	8.0 ± 2.4	6.2 ± 1.8	<0.001
Reasons for stopping exercise – %					0.628
Dyspnea	24	29	29	44	
Leg fatigue	47	29	41	25	
Dyspnea and leg fatigue	12	18	6	19	
Other	17	24	24	12	

Plus-minus values are mean ± SD and values with parentheses are median (25^th^, 75^th^ percentile). GOLD 1–4 defined by percent predicted FEV_1_, and GOLD group A-D defined by symptoms and exacerbation risk (see Methods for details). P-values computed with percent emphysema as a continuous variable.

Abbreviations: HU = Hounsfield units; mMRC = modified Medical Research Council dyspnea scale; FEV_1_ forced expired volume in 1-sec; FVC = forced vital capacity; GOLD = Global Initiative for Chronic Obstructive Lung Disease; TLC = total lung capacity; D_LCO_ = diffusing capacity for carbon monoxide; ${\dot{{\rm{V}}}O}_{2}$ = rate of O_2_ uptake; ${\dot{{\rm{V}}}}_{{\rm{E}}}$ = minute ventilation; V_T_ = tidal volume; ${\dot{{\rm{V}}}\text{CO}}_{2}$ = rate of CO_2_ output; ${\dot{{\rm{V}}}}_{{\rm{E}}}$/${\dot{{\rm{V}}}\text{CO}}_{2}$ = ventilatory equivalent for CO_2_; P_ET_CO_2_ = end-tidal partial pressure of CO_2_; S_p_O_2_ = pulse-oximeter estimate oxy-hemoglobin saturation.

### Emphysema and peak exercise capacity

**Figure 1:**
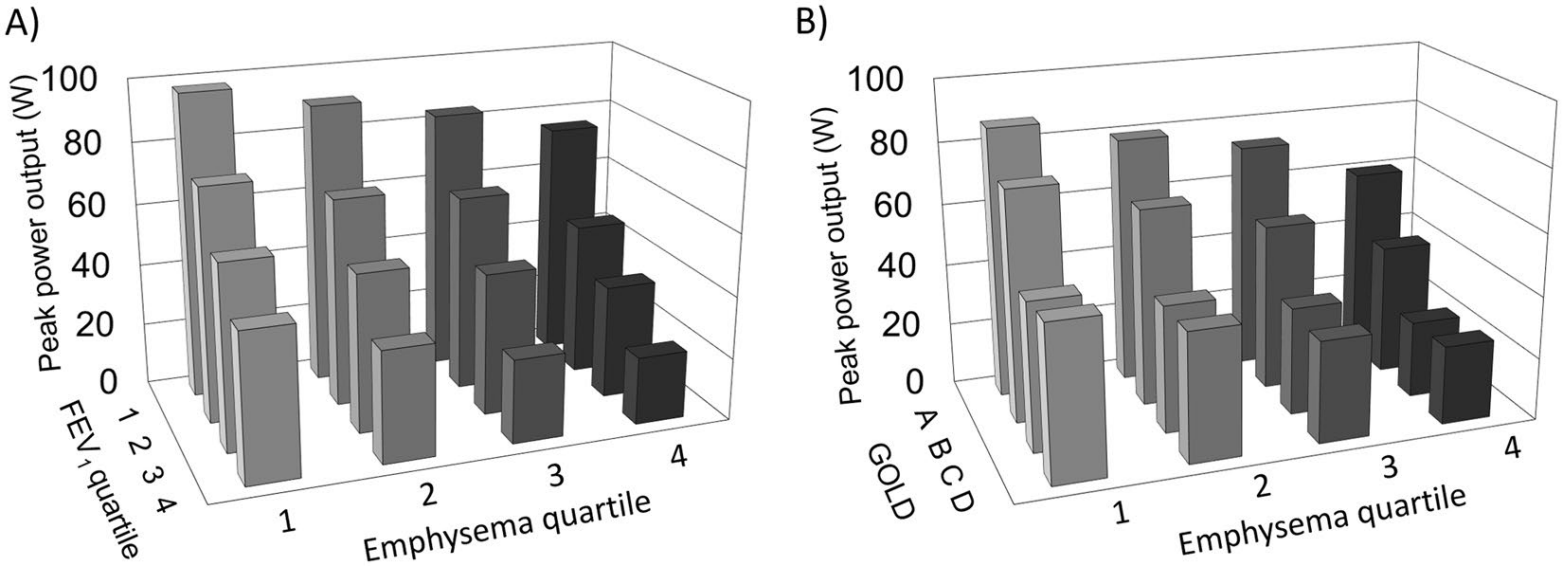
Percent emphysema was associated with peak exercise capacity independent of airflow limitation severity, and symptom burden/exacerbation frequency. Peak power output-percent emphysema relationship stratified by airflow limitation (panel A), and GOLD A–D (panel B). To account for potential confounders, peak power output was calculated using linear regression to adjust for age, gender, height, body mass index, depth of inspiration at CT, smoking status, and FEV_1_ percent predicted (panel A) or GOLD group A–D (panel B). GOLD group A–D was defined by symptom burden and exacerbation frequency (See Methods for details). Abbreviations: FEV_1_ = forced expired volume in one second; COPD = chronic obstructive pulmonary disease; CT = computed tomography; and GOLD = Global Initiative for Chronic Obstructive Lung Disease.

PPO and ${\dot{{\rm{V}}}O}_{2}$_Peak_ were 61 ± 32 W (47 ± 27% predicted) and 12 ± 5 ml/kg/min (57 ± 30% predicted), respectively. Independent of airflow limitation severity (GOLD 1–4), higher percent emphysema was associated with lower PPO (−24 W per 10% increment in emphysema; 95%CI: −41 to −7), and lower ${\dot{{\rm{V}}}O}_{2}$_Peak_ (−2.7 ml/kg/min per 10% increment in emphysema; 95% CI: −5.2 to −0.2), and these associations remained significant in models adjusting for FEV_1_ as a continuous variable, and for GOLD group A-D (Fig. 1 and Table 2). Similar results were obtained with additional adjustment for pectoralis muscle area or pulmonary artery-to-aorta diameter (p ≤ 0.005).

**Table 2:** Relationship between percent emphysema, peak exercise capacity, peak perceptual responses, and ventilatory inefficiency, expiratory flow limitation and inspiratory neural drive throughout incremental cycle exercise in chronic obstructive pulmonary disease.

	Mean difference in exercise response per 10% increment in percent emphysema (95% CI)				
	Unadjusted	Model 1	Model 1 + GOLD 1–4	Model 1 + FEV_1_ percent predicted	Model 1 + GOLD A-D
Peak power output – W	**−29 (−46 to −12) P < 0**.**001**	**−41 (−59 to −24) P < 0**.**001**	**−24 (−41 to −7) P = 0**.**007**	**−21 (−34 to −9) P = 0**.**001**	**−31 (−47 to −14) P < 0**.**001**
Peak ${\dot{{\rm{V}}}O}_{2}$ – mL/kg/min	**−4**.**3 (−6**.**6 to −1**.**9) P < 0**.**001**	**−6**.**2 (−8**.**8 to −3**.**6) P < 0**.**001**	**−2**.**7 (−5**.**2 to −0**.**2) P = 0**.**036**	**−2**.**3 (−4**.**6 to −0**.**1) P = 0**.**038**	**−4**.**6 (−7**.**3 to −1**.**9) P < 0**.**001**
Peak dyspnea/ ${\dot{{\rm{V}}}}_{{\rm{E}}}$ ratio – Borg units/L/min	**0**.**04 (0**.**01 to 0**.**07) P = 0**.**021**	**0**.**05 (0**.**01 to 0**.**08) P = 0**.**006**	0.01 (−0.03 to 0.04) P = 0.682	0.01 (−0.10 to 0.05) P = 0.721	**0**.**03 (0**.**0 to 0**.**06) P = 0**.**022**
Peak leg fatigue/ ${\dot{{\rm{V}}}O}_{2}$ ratio – Borg units/mL/kg/min	0.93 (−0.67 to 2.53) P = 0.253	1.19 (−0.65 to 3.03) P = 0.206	0.45 (−1.40 to 2.30) P = 0.632	0.41 (−1.45 to 2.28) P = 0.663	0.73 (−0.82 to 2.27) P = 0.356

Mean differences in exercise responses at peak exercise estimated by linear regression. Model 1 adjusts for age, gender, height, body mass index, depth of inspiration at CT, and smoking status. GOLD 1–4 defined by strata of percent predicted FEV_1_, and GOLD group A-D defined by symptoms and exacerbation risk.

Abbreviations: CI = confidence interval; ${\dot{{\rm{V}}}O}_{2}$ = rate of O_2_ uptake; ${\dot{{\rm{V}}}}_{{\rm{E}}}$ = minute ventilation; CT = computed tomography; FEV_1_ = forced expired volume in 1-sec; and GOLD = Global Initiative for Chronic Obstructive Lung Disease.

### Emphysema and perceptual responses at peak exercise

Higher dyspnea intensity-${\dot{{\rm{V}}}}_{{\rm{E}}}$ and leg fatigue-${\dot{{\rm{V}}}O}_{2}$ ratios at peak exercise were observed with higher percent emphysema; however, these associations were not significant after accounting for airflow limitation severity (Table 2).The reasons for stopping exercise were not associated with percent emphysema after adjusting for airflow limitation (p > 0.16 for dyspnea, leg discomfort or other). Similar results were obtained with additional adjustment for pectoralis muscle area or pulmonary artery-to-aorta diameter (p ≥ 0.503 for all analyses).

### Emphysema and the cardiorespiratory responses throughout exercise

**Figure 2:**
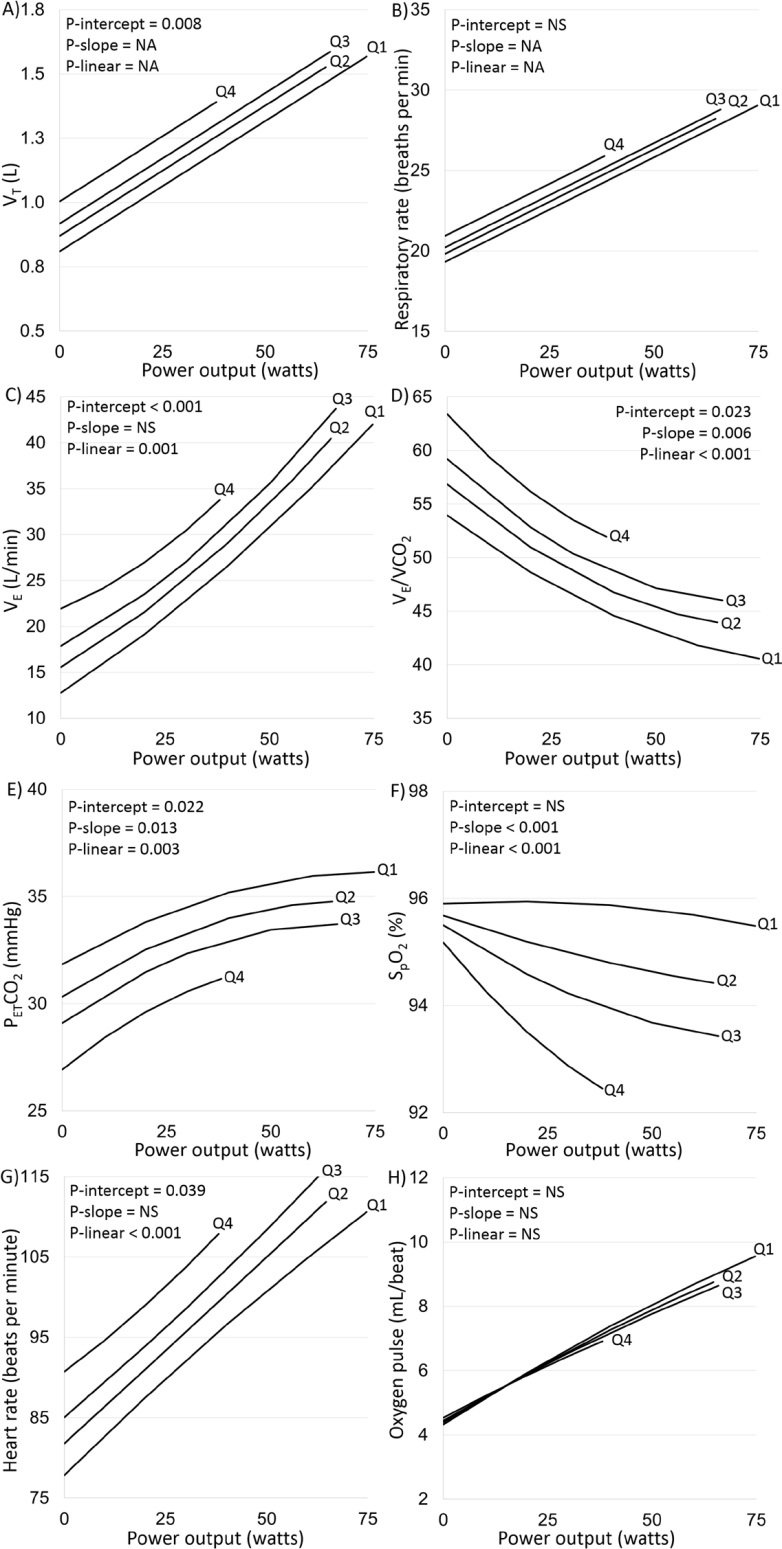
Cardiorespiratory responses to symptom-limited incremental cycle exercise testing by quartile of percent emphysema independent of airflow limitation. Each panel depicts the relationship between percent emphysema quartile (Q1: 3.1%; Q2: 8.4%; Q3: 14.5%; Q4: 27.5%) and a cardiorespiratory response (Y-axis) throughout exercise (X-axis). Curves were derived from mixed model regression adjusting for age, gender, height, body mass index, depth of inspiration at CT, smoking status, and airflow limitation severity (GOLD 1–4). P-intercept is the probability that percent emphysema predicts no difference in cardiorespiratory response at the intercept (i.e., rest). P-slope is the probability that percent emphysema predicts no difference in slope between exercise intensity and cardiorespiratory response. P-linear is the probability that the percent emphysema association with the cardiorespiratory response is linear. NA denotes the model did not require a slope or nonlinear term for optimum fit (See Methods for details). Abbreviations: V_T_ = tidal volume; ${\dot{{\rm{V}}}O}_{2}$ = rate of O_2_ uptake; ${\dot{{\rm{V}}}}_{{\rm{E}}}$ = minute ventilation; ${\dot{{\rm{V}}}\text{CO}}_{2}$ = rate of CO_2_ output; P_ET_CO_2_ = end-tidal partial pressure of CO_2_; S_p_O_2_ = pulse-oximeter estimated arterial oxy-hemoglobin saturation; CT = computed tomography; and GOLD = Global Initiative for Chronic Obstructive Lung Disease; NA = not applicable.

Throughout exercise and independent of airflow limitation severity (GOLD 1–4), percent emphysema was associated with higher V_T_, similar respiratory rate, higher V_E_, higher $\dot{{\rm{V}}}E$-${\dot{{\rm{V}}}\text{CO}}_{2}$ slope, lower P_ET_CO_2_, lower S_p_O_2_, higher heart rate, and lower O_2_ pulse (Fig. 2). Similar associations were observed with ${\dot{{\rm{V}}}O}_{2}$ as the measure of exercise intensity (e-Fig. 1), and when adjusting for FEV_1_ as a continuous variable (e-Fig. 1), or GOLD A-D (e-Fig. 2).

Percent emphysema was associated with higher ${\dot{{\rm{V}}}}_{{\rm{E}}}$-${\dot{{\rm{V}}}\text{CO}}_{2}$ slope during exercise in unadjusted and adjusted analyses including spirometric GOLD 1–4 (Table 3). Percent emphysema was associated with higher ${\dot{{\rm{V}}}}_{{\rm{E}}}$/${\dot{{\rm{V}}}\text{CO}}_{2}$ nadir independent of airflow limitation severity (GOLD 1–4 or FEV_1_ as a continuous variable) and GOLD group A-D, and which occurred at a lower exercise intensity and with lower P_ET_CO_2_ (e-Table 1).

**Table 3:** Relationship between percent emphysema and measures of ventilatory inefficiency, expiratory flow limitation and inspiratory neural drive throughout incremental cycle exercise testing in chronic obstructive pulmonary disease.

	Mean difference in exercise response per 10% increment in percent emphysema (95% CI)				
	Unadjusted	Model 1	Model 1 + GOLD 1–4	Model 1 + FEV_1_ percent predicted	Model 1 + GOLD A-D
${\dot{{\rm{V}}}}_{{\rm{E}}}$/${\dot{{\rm{V}}}\text{CO}}_{2}$ slope	**5**.**9 (1**.**0 to 10**.**8) p = 0**.**019**	**8**.**1 (1**.**5 to 14**.**6) p = 0**.**015**	**16**.**8 (9**.**3 to 24**.**4) P < 0**.**001**	**17**.**1 (9**.**5 to 24**.**7) P < 0**.**001**	**8**.**7 (2**.**6 to 14**.**8) P = 0**.**005**
${\dot{{\rm{V}}}}_{{\rm{T}}}$/T_E_ − ${\dot{{\rm{V}}}}_{{\rm{E}}}$ slope – mL/sec/L/min	**−4**.**2 (−6**.**0 to −2**.**4) P < 0**.**001**	**−3**.**8 (−6**.**0 to −1**.**6) P < 0**.**001**	−0.9 (−3.5 to 1.7) P = 0.500	−0.7 (−3.2 to 1.8) P = 0.575	**−3**.**4 (−5**.**8 to −1**.**1) P = 0**.**004**
${\dot{{\rm{V}}}}_{{\rm{T}}}$/T_I_ − ${\dot{{\rm{V}}}}_{{\rm{E}}}$ slope – mL/sec/L/min	**12**.**3 (7**.**2 to 17**.**4) P < 0**.**001**	**11**.**9 (5**.**8 to 18**.**0) P < 0**.**001**	3.2 (−3.6 to 10.1) P = 0.350	2.9 (−3.8 to 9.6) P = 0.391	**10**.**9 (4**.**8 to 17**.**0) P < 0**.**001**

Mean differences in exercise responses estimated by linear regression. Model 1 adjusts for age, gender, height, body mass index, depth of inspiration at CT, and smoking status. GOLD 1–4 defined by percent predicted FEV_1_, and GOLD group A-D defined by symptoms and exacerbation risk (see Methods for details).

Abbreviations: CI = confidence interval; ${\dot{{\rm{V}}}}_{{\rm{E}}}$ = minute ventilation; ${\dot{{\rm{V}}}\text{CO}}_{2}$ = rate of CO_2_ output; V_T_ = tidal volume; T_E_ = expiratory time; T_I_ = inspiratory time; V_T_/_TE_ = mean tidal expiratory flow rate; V_T_/T_E_ − ${\dot{{\rm{V}}}}_{{\rm{E}}}$ slope = crude estimate of expiratory flow limitation, where a lower V_T_/T_E_ − ${\dot{{\rm{V}}}}_{{\rm{E}}}$ slope reflects greater expiratory flow limitation; V_T_/T_I_ = mean tidal inspiratory flow rate; V_T_/T_I_ − ${\dot{{\rm{V}}}}_{{\rm{E}}}$ slope = crude estimate of inspiratory neural drive, where a higher V_T_/T_I_ − ${\dot{{\rm{V}}}}_{{\rm{E}}}$ slope reflects greater inspiratory neural drive; CT = computed tomography; FEV_1_ = forced expired volume in 1-sec; and GOLD = Global Initiative for Chronic Obstructive Lung Disease.

Slopes of the linear relationships between exercise-induced increases in ${\dot{{\rm{V}}}}_{{\rm{E}}}$ and each of V_T_/T_I_ and V_T_/T_E_ were also associated with percent emphysema, but not after adjustment for GOLD 1–4 or FEV_1_ as a continuous variable (Table 3).

Similar results were obtained with additional adjustment for pectoralis muscle area or pulmonary artery-to-aorta diameter (e-Fig. 3 and 4).

## Discussion

Among smokers with COPD, the extent of pulmonary emphysema was associated with reduced exercise capacity that was independent of standard measures of disease severity, including spirometric airflow limitation, symptom burden (mMRC dyspnea score) and exacerbation risk. Throughout exercise, emphysema was also independently associated with ventilatory inefficiency, impaired gas-exchange, and increased heart rate despite similar dyspnea and leg discomfort ratings and reasons for stopping exercise. This is important considering that obstructive changes and parenchymal destruction (emphysema) will vary from person to person, and they could evolve at different rates over time. These observations suggest that, independent of the severity of airflow limitation, emphysema contributes significantly to exercise intolerance, and may not respond to COPD therapies targeting bronchoconstriction and airways inflammation, particularly among adults with emphysema-predominant COPD.

To our knowledge the present study is the first to investigate the impact of emphysema severity at CT on the physiological and perceptual response to incremental exercise independent of contemporary measures of disease severity and symptom burden^1^. Emphysema assessed visually^20^ and quantitatively^24^ by CT have been correlated with lower ${\dot{{\rm{V}}}O}_{2}$_Peak_, and peak S_p_O_2_ on incremental treadmill exercise testing. Similarly, correlations have been described between emphysema severity and six-minute walk distance^18,19,25–29^. The present study supports and builds upon these observations by demonstrating that differences in exercise capacity are independent of currently recommended measures of COPD severity, symptom burden and exacerbation risk^1^. These observations suggest that emphysema severity, readily assessed at CT, is a potential indicator (“biomarker”) of physiological impairment in COPD that is unlikely to respond to therapies targeting airflow limitation alone.

The mechanisms of emphysema-associated exercise intolerance in COPD are incompletely understood and likely multi-factorial. Crisafulli, Jones, and Paoletti each reported emphysema-associated exercise ventilatory inefficiency^21,22,24^. Consistent with these observations, an early study of ventilation-perfusion in advanced COPD demonstrated that virtually all subjects with Burrows type A (emphysematous) COPD had high ventilation-perfusion ratios as compared with type B (bronchial) or mixed COPD phenotypes^30,31^. Notably, the pattern of ventilation-perfusion inequality in that study was not associated with the degree of spirometric impairment^30^. More recently, studies have reported an emphysematous phenotype of COPD with significant pulmonary hypertension, hypoxemia, and hypocapnia, despite only mild-to-moderate airflow limitation assessed by spirometry^32,33^. Furthermore, cardiac magnetic resonance studies in COPD have demonstrated lower lung perfusion and cardiac under filling with emphysema^14–17^. Emphysematous COPD is associated with higher levels of sarcopenia, which also contributes to exercise intolerance^34,35^. Our study builds upon these observations by showing that emphysema contributes to significant exercise impairment independent of currently recommended disease severity measures (spirometry, symptom burden, and exacerbation risk) in mild-to-very severe COPD. We further demonstrate emphysema-specific cardiorespiratory exercise responses (ventilatory inefficiency, impaired gas-exchange and cardiac response) independent of COPD severity, pulmonary arterial enlargement, and pectoralis muscle area^36,37^, and despite similar ratings of dyspnea and leg discomfort.

Together, the novel results of our study i) suggest that emphysema adds to the endotypic characterization of impairment in COPD; ii) strengthens evidence for mechanisms of emphysema-induced exercise that appear to be independent of established mechanisms of airflow limitation, pulmonary hypertension, and sarcopenia; iii) highlight the need for therapeutic targets beyond airways disease^38–40^; iv) and may inform participant selection (endotypic medicine) for clinical trials targeting the pathobiology of emphysematous COPD^8^.

We speculate that emphysema-specifc pathophysiological abnormalities in pulmonary gas exchange with attendant arterial blood O_2_ desaturation (as indicated by the SpO_2_ findings), cardiac dysfunction (as indicated by heart response findings), and unmeasured arterial hypoxemia with or without arterial hypercapnia and respiratory acidosis would impair exercise tolerance by compromising peripheral locomotor muscle O_2_ delivery, accelerating the rate of peripheral locomotor muscle fatigue development and increasing central respiratory motor drive via increased stimulation of central and peripheral chemoreceptors and perhaps also muscle metaboreceptors (type IV sensory afferents)^41,42^. Clinical physiology studies with detailed assessments of arterial blood gases and peripheral locomotor muscle function are needed to substantiate this hypothesis.

The present study has limitations. First, potential mechanisms of emphysema-associated exercise intolerance were not assessed, including the behavior of dynamic operating lung volumes (e.g., dynamic lung hyperinflation), dead space, hemodynamics (central, peripheral and pulmonary), and peripheral muscle dysfunction. Nevertheless, the current findings, to the best of our knowledge, represent the first demonstration of emphysema-associated exercise impairment that is independent of contemporary measures of COPD severity, symptom burden and exacerbation risk, and justify future investigations into the mechanism(s) of this association. Second, non-smokers and participants without COPD were not included in the study sample. We believe this design strategy limited the heterogeneity of disease pathogenesis and permitted a focused investigation of emphysema severity in clinical COPD. Third, our indirect measures of pulmonary arterial pressure, muscle wasting, as well as retrospective exacerbation frequency may have left residual confounding. Future analysis with invasive and prospective measures are needed, in addition to samples with greater muscle wasting. Finally, the sample size was modest and limited to a clinical population of smokers, three-quarters of whom were men, potentially limiting generalizability. However, patients were selected across the range of severity of airflow limitation (GOLD 1–4) and symptom burden/exacerbation risk (GOLD A-D). While our use of mixed model regression leveraged all observed data points, thereby reducing selection bias, increasing precision and accounting for auto-correlations, further study is needed in smokers without airflow limitation having structural evidence of lung disease manifested by the varying presence of emphysema, and among non-smokers.

In summary, in a clinical sample of COPD patients with past or current smoking and mild-to-very-severe airflow limitation, CT-quantified emphysema is associated with exercise intolerance, ventilatory inefficiency, impaired gas-exchange, and evidence of exaggerated heart rate response that is independent of airflow limitation, symptom burden, and exacerbation risk. The novel results of our study (1) suggest that emphysema endotyping may add to the multi-dimensional characterization of COPD currently recommended by GOLD, and (2) highlight the need for clinical research targeting emphysema-associated pathophysiology beyond airflow limitation.

## Methods

Adults with clinically stable COPD were recruited from the outpatient department of the Montreal Chest Institute. Included participants were 40–79 years of age with at least 10 pack-years of smoking history. Exclusion criteria were exacerbation or change in COPD medication in the preceding 6 weeks, other physician-diagnosed lung disease (any history of asthma, tuberculosis, cancer, cystic fibrosis, or transplant), congestive heart failure, or any other disease considered to be a contraindication to study participation by the treating physician.

The McGill University Health Centre review board approved the study protocol (BMC-7-011). Written informed consent was obtained from all participants. All procedures were performed in accordance with the relevant guidelines and regulations.

### Emphysema assessment

Full-lung thoracic CT scans were acquired at suspended maximal inspiration after participant coaching on a single General Electric helical 16 multi-slice scanner using a standardized protocol (matrix 512 × 512; 120 kVp; 40 mA; slice thickness 1.25 mm; pitch 1.375:1). Quantitative CT analysis was performed using the Pulmonary Workstation 2.0 software package (VIDA Diagnostics, Inc., Coralville, IA). The lungs were automatically segmented from the bronchial tree and surrounding chest wall and mediastinal components. The lung volume was calculated from the segmented images. The percent of emphysema-like lung was defined as the percent of lung voxels below −950 Hounsfield units, hereafter referred to as percent emphysema. The depth of inspiration at CT, which is associated with lung density^43^, was defined as the ratio of lung volume achieved at CT-to-plethysmographic total lung capacity.

### Pulmonary function testing

Post-bronchodilator spirometry, body plethysmography, and single breath diffusing capacity for carbon monoxide (D_LCO_) were performed according to current standards^44–46^. Predicted spirometry, lung volume and D_LCO_ values were calculated from reference equations^47–49^.

COPD status and airflow limitation severity were defined according to the 2017 Global Initiative for Chronic Obstructive Lung Disease (GOLD) strategy^1^. All participants had a post-bronchodilator forced expired volume in 1-sec-to-forced vital capacity ratio (FEV_1_/FVC) below 0.70^1^. GOLD spirometric grades were defined by FEV_1_ percent-predicted (GOLD 1: ≥ 80%; 2: 50–79%; 3: 30–49%; 4: < 30%), and GOLD group by the modified Medical Research Council (mMRC) dyspnea rating and exacerbation frequency in the preceding 12 months (GOLD A: mMRC < 2, and exacerbations < 2 with no hospitalization; B: mMRC ≥ 2 and exacerbations < 2 with no hospitalization; C: mMRC < 2, and exacerbations ≥ 2 or hospitalization; D: mMRC ≥ 2 and exacerbations ≥ 2 or hospitalization)^1^.

### Exercise testing

Symptom-limited incremental exercise tests were performed on an electronically-braked cycle ergometer (Vmax Encore^TM^, CareFusion) according to guidelines^50^. After a rest period of at least 6-min, participants performed 1-min of unloaded pedaling (warm-up), followed by stepwise increases in power output (10 W/min) until symptom limitation. Standard cardiopulmonary parameters were collected breath-by-breath, while oxy-hemoglobin saturation and heart rate were monitored by pulse-oximetry (S_p_O_2_) and 12-lead electrocardiogram, respectively. Intensity ratings of dyspnea and leg fatigue were assessed using Borg’s modified 0–10 category ratio scale^51^ at rest and the symptom-limited peak of exercise. Participants also verbalized their main reason(s) for stopping exercise. Peak power output (PPO) and oxygen uptake (${\dot{{\rm{V}}}O}_{2}$_Peak_) were defined as the average of the last 30-sec of loaded pedaling. Predicted PPO and ${\dot{{\rm{V}}}O}_{2}$_Peak_ were calculated from reference equations^52^. Dyspnea intensity divided by ${\dot{{\rm{V}}}}_{{\rm{E}}}$ (dyspnea intensity-${\dot{{\rm{V}}}}_{{\rm{E}}}$), and leg fatigue divided by ${\dot{{\rm{V}}}O}_{2}$ (leg fatigue-${\dot{{\rm{V}}}O}_{2}$) at peak exercise were also calculated.

Throughout exercise ${\dot{{\rm{V}}}O}_{2}$, tidal volume (V_T_), respiratory rate, minute ventilation (${\dot{{\rm{V}}}}_{{\rm{E}}}$), inspiratory and expiratory times (T_I_, T_E_, respectively), end-tidal partial pressure of carbon dioxide (P_ET_CO_2_), S_p_O_2_, and O_2_ pulse (${\dot{{\rm{V}}}O}_{2}$ divided by heart rate) were averaged over the last 30-sec of every 10 W interval of exercise. The slopes of ${\dot{{\rm{V}}}}_{{\rm{E}}}$ versus carbon dioxide output (${\dot{{\rm{V}}}}_{{\rm{E}}}$ − ${\dot{{\rm{V}}}\text{CO}}_{2}$ slope), V_T_ divided by T_E_ versus ${\dot{{\rm{V}}}}_{{\rm{E}}}$ (V_T_/T_E_- ${\dot{{\rm{V}}}}_{{\rm{E}}}$ slope), and V_T_ divided by T_I_ versus ${\dot{{\rm{V}}}}_{{\rm{E}}}$ (V_T_/T_I_ − ${\dot{{\rm{V}}}}_{{\rm{E}}}$ slope) were calculated as crude estimates of exercise ventilatory efficiency, inspiratory neural drive, and expiratory flow limitation, respectively. The ${\dot{{\rm{V}}}}_{{\rm{E}}}$/${\dot{{\rm{V}}}\text{CO}}_{2}$ nadir was defined as the lowest 30-sec average data point observed during symptom-limited incremental exercise.

### Covariables

Body height and mass were measured by standardized protocol and body mass index (BMI) calculated as the weight in kilograms divided by height in meters squared. Gender, smoking status, pack-years of smoking, mMRC dyspnea rating, and exacerbation frequency and severity (admission vs no admission) in the prior 12-months were assessed via standardized questionnaire. Axial CT scan images were used to visually map and quantify pectoralis muscle area at the superior aspect of the aortic arch^36^, and the pulmonary artery and aorta diameters at the level of the main pulmonary artery bifurcation^37^.

### Statistical analysis

Continuous variables are presented as mean ± SD and dichotomous variables as proportions unless otherwise indicated. Percent emphysema was square-root transformed for all regression analyses to obtain normally distributed residuals.

Peak exercise capacity measures (PPO, ${\dot{{\rm{V}}}O}_{2}$_Peak_) were modeled using linear regression with percent emphysema as the predictor of interest. Models were adjusted for age, gender, height, BMI, smoking status, depth of inspiration at CT, smoking status, and airflow limitation severity (GOLD 1–4). The same approach was used to model peak dyspnea intensity-${\dot{{\rm{V}}}}_{{\rm{E}}}$ and leg fatigue-${\dot{{\rm{V}}}O}_{2}$ ratios.

The impact of percent emphysema on cardiorespiratory exercise responses were assessed by mixed model regression. This analytic approach permits inclusion of all observed time points despite between-subject differences in peak exercise capacity (thus maximizing precision), accounts for within-subject correlations of repeated measures, and allows for covariable adjustment including measures of COPD severity. The multivariable models included the covariables listed above, and an interaction term between percent emphysema and exercise intensity measure. Linear and non-linear ${\dot{{\rm{V}}}O}_{2}$ terms were explored, and model selection was based on the model with the lowest Akaike information criterion value for each cardiorespiratory response.

Sensitivity analyses adjusted for FEV_1_ percent predicted as a continuous variable, and GOLD group A-D. Post hoc analyses additionally adjusted for pectoralis muscle area^36^, and the ratio of pulmonary artery diameter to aorta diameter^37^. All analyses were performed using SAS 9.4 (Cary, NC). A p-value less than 0.05 was considered statistically significant.

## Electronic supplementary material

Supplementary Information
