# Supplementary material for: Impact of pulmonary emphysema on exercise capacity and its physiological determinants in chronic obstructive pulmonary disease
Source: Sci Rep. 2018 Oct 24;8:15745. doi: 10.1038/s41598-018-34014-5 (PMC6200804; doi:10.1038/s41598-018-34014-5)
Supplement: Supplementary file 1 — Supplementary Information [file 41598_2018_34014_MOESM1_ESM.docx]

**e-APPENDIX TITLE PAGE**

**Title:** Impact of pulmonary emphysema on exercise capacity and its physiological determinants in COPD

**Authors**:

Benjamin M Smith MD MS^1,2,3,4,*^

Dennis Jensen PhD^1,3^

Marc Brosseau MD^1^

Andrea Benedetti PhD^1,4^

Harvey O Coxson PhD^5^

Jean Bourbeau MD MS^1,4^

**Affiliations**:

1 McGill University Health Centre Research Institute, Montreal, Canada

2 Columbia University Medical Center, New York, USA

3 Department of Kinesiology and Physical Education, McGill University, Montreal, Canada

4 Department of Epidemiology, Biostatistics and Occupational Health, McGill University, Montreal, Canada

5 Department of Radiology, University of British Columbia, Canada

**e-Figures**: Page 2

**e-Table**: Page 7

**e-Figure 1**. Cardiorespiratory responses to symptom-limited incremental cycle exercise by quartile of percent emphysema independent of spirometric airflow limitation with rate of oxygen uptake as the measure of exercise intensity.

**
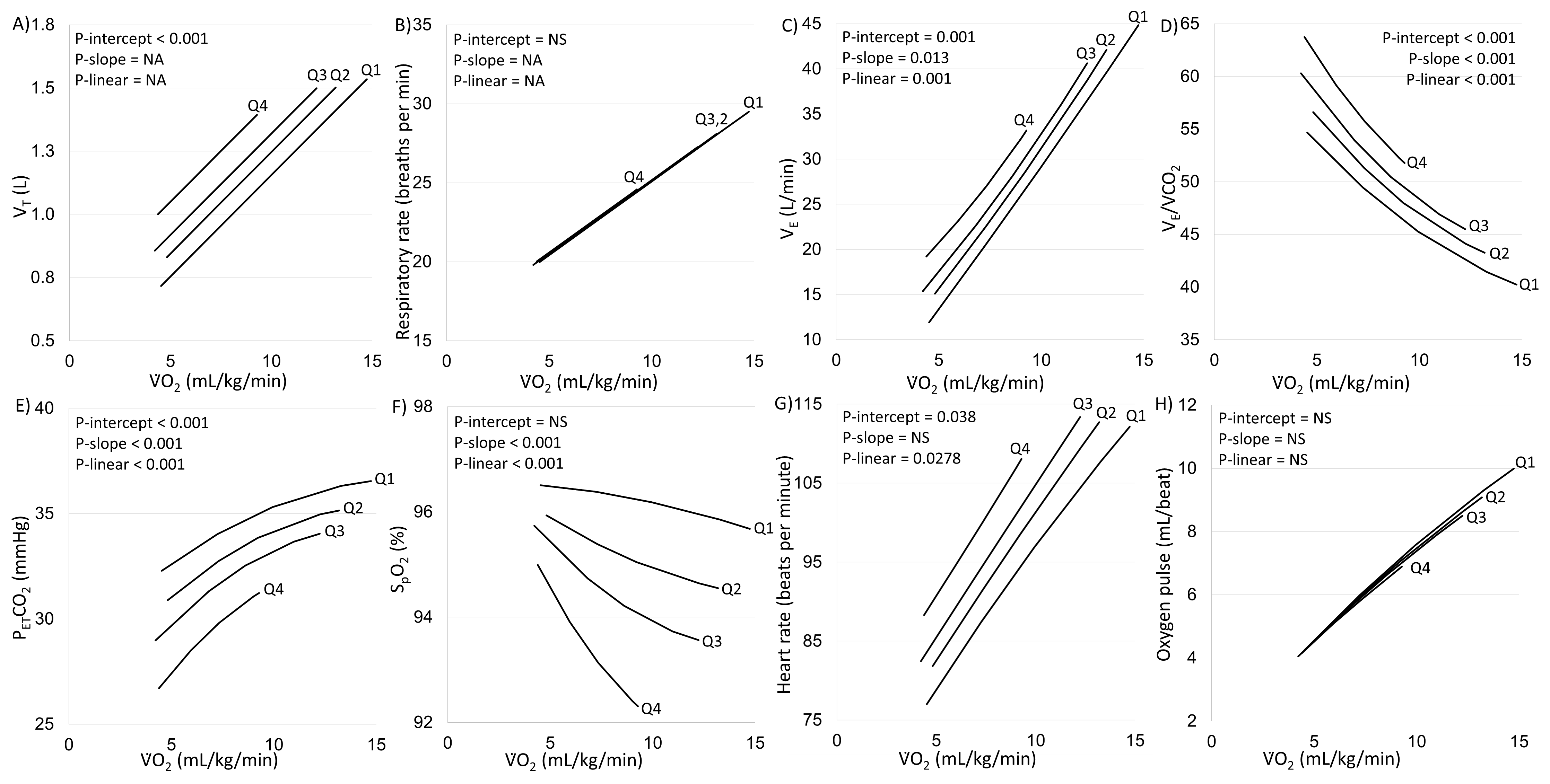
**

Each panel shows the continuous relationship between percent emphysema (depicted as quartiles: Q1: 3.1%; Q2: 8.4%; Q3: 14.5%; Q4: 27.5%) and a cardiorespiratory response (Y-axis) throughout exercise (X-axis). Curves were derived from mixed model regression to adjust for age, gender, BMI, depth of inspiration at CT, smoking status, and percent predicted FEV_1_. P-intercept is the probability that percent emphysema does predicts no difference in cardiorespiratory response at the intercept. P-slope is the probability that percent emphysema predicts no difference in slope between exercise intensity and cardiorespiratory response. P-linear is the probability that the percent emphysema association with the cardiorespiratory response is linear. NA denotes the model did not require a slope or nonlinear term for optimum fit (See Methods for details). Abbreviations: V̇O_2_ = rate of O_2_ uptake; V̇_E_ = minute ventilation; V̇CO_2_ = rate of CO_2_ output; P_ET_CO_2_ = end-tidal partial pressure of CO_2_; S_p_O_2_ = pulse-oximeter estimated arterial oxy-hemoglobin saturation; CT = computed tomography; and GOLD = Global Initiative for Chronic Obstructive Lung Disease; NA = not applicable; and BMI = body mass index.

**e-Figure 2**. Cardiorespiratory responses to incremental exercise by quartile of percent emphysema independent of GOLD A-D.


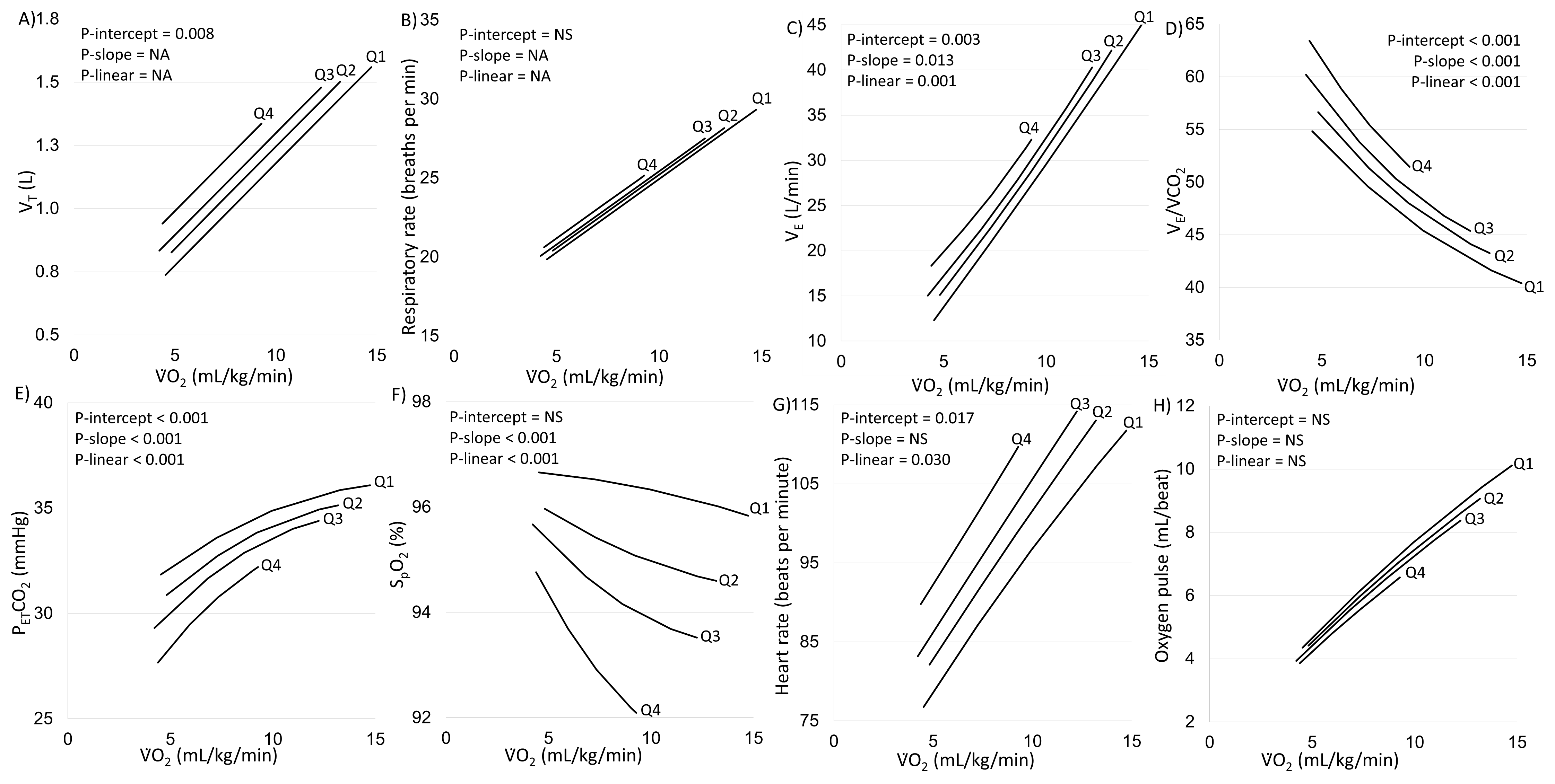


Each panel shows the relationship between a cardiorespiratory response (Y-axis) throughout exercise (X-axis) and percent emphysema (depicted by quartile: Q1: 3.1%; Q2: 8.4%; Q3: 14.5%; Q4: 27.5%). Curves were derived from mixed model regression adjusted for age, gender, BMI, depth of inspiration at CT, smoking status, and GOLD A-D. P-intercept is the probability that percent emphysema predicts no difference in cardiorespiratory response at the intercept. P-slope is the probability that percent emphysema predicts no difference in slope between exercise intensity and cardiorespiratory response. P-linear is the probability that the percent emphysema association with the cardiorespiratory response is linear. NA denotes the model did not require a slope or nonlinear term for optimum fit (See Methods for details). Abbreviations: V_T_ = tidal volume; V̇O_2_ = rate of O_2_ uptake; V̇_E_ = minute ventilation; V̇CO_2_ = rate of CO_2_ output; P_ET_CO_2_ = end-tidal partial pressure of CO_2_; S_p_O_2_ = pulse-oximeter estimated arterial oxy-hemoglobin saturation; CT = computed tomography; and NA = not applicable; and BMI = body mass index.

**e-Figure 3**. Cardiorespiratory responses to incremental exercise by quartile of percent emphysema independent of pectoralis muscle area.


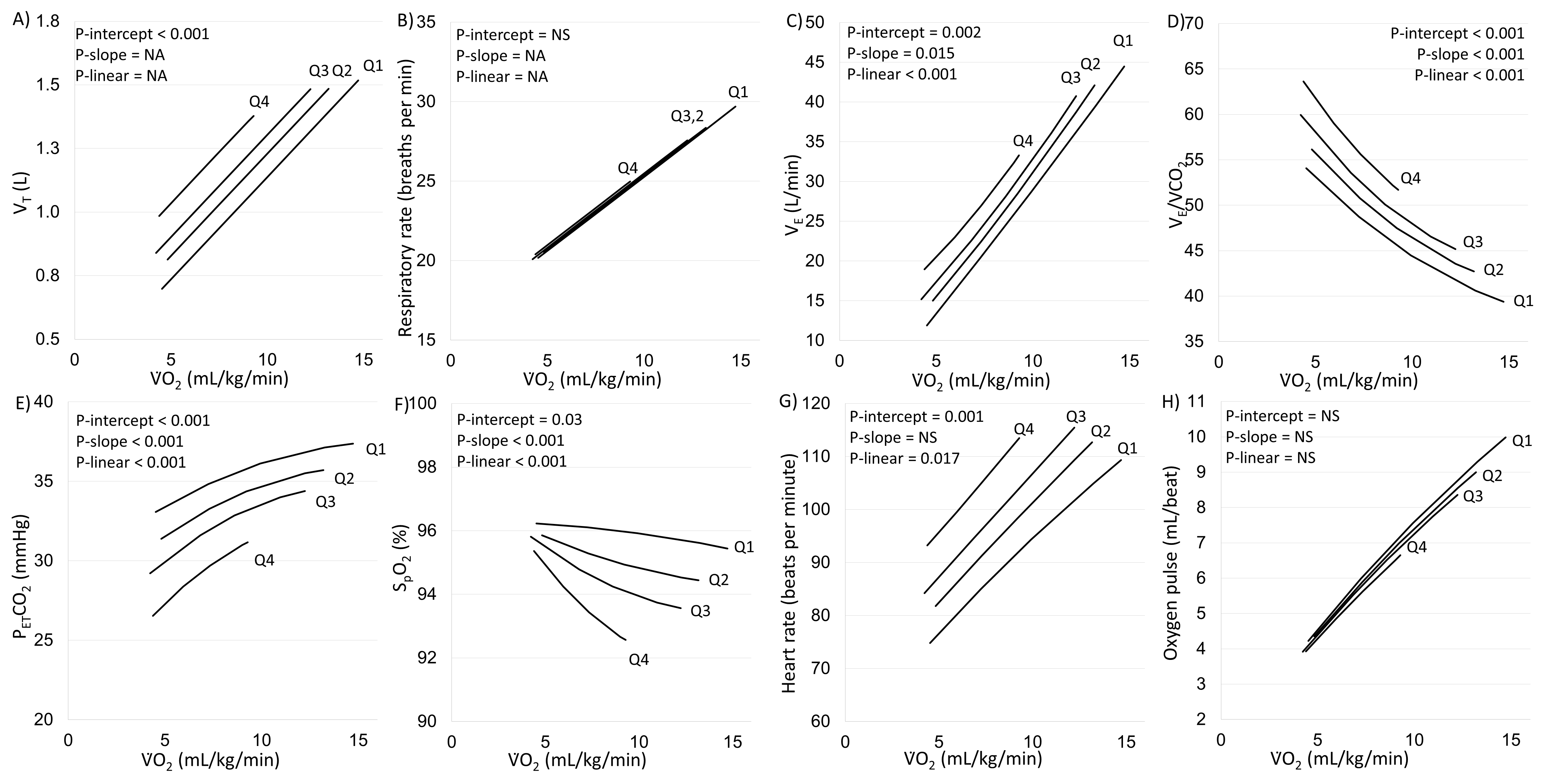


Each panel shows the relationship between a cardiorespiratory response (Y-axis) throughout exercise (X-axis) and percent emphysema (depicted by quartile: Q1: 3.1%; Q2: 8.4%; Q3: 14.5%; Q4: 27.5%). Curves were derived from mixed model regression adjusted for age, gender, BMI, depth of inspiration at CT, smoking status, and pectoralis muscle area. P-intercept is the probability that percent emphysema predicts no difference in cardiorespiratory response at the intercept. P-slope is the probability that percent emphysema predicts no difference in slope between exercise intensity and cardiorespiratory response. P-linear is the probability that the percent emphysema association with the cardiorespiratory response is linear. NA denotes the model did not require a slope or nonlinear term for optimum fit (See Methods for details). Abbreviations: V_T_ = tidal volume; V̇O_2_ = rate of O_2_ uptake; V̇_E_ = minute ventilation; V̇CO_2_ = rate of CO_2_ output; P_ET_CO_2_ = end-tidal partial pressure of CO_2_; S_p_O_2_ = pulse-oximeter estimated arterial oxy-hemoglobin saturation; CT = computed tomography; and NA = not applicable; and BMI = body mass index.

**e-Figure 4**. Cardiorespiratory responses to incremental exercise by quartile of percent emphysema independent of pulmonary artery-to-aorta diameter ratio.


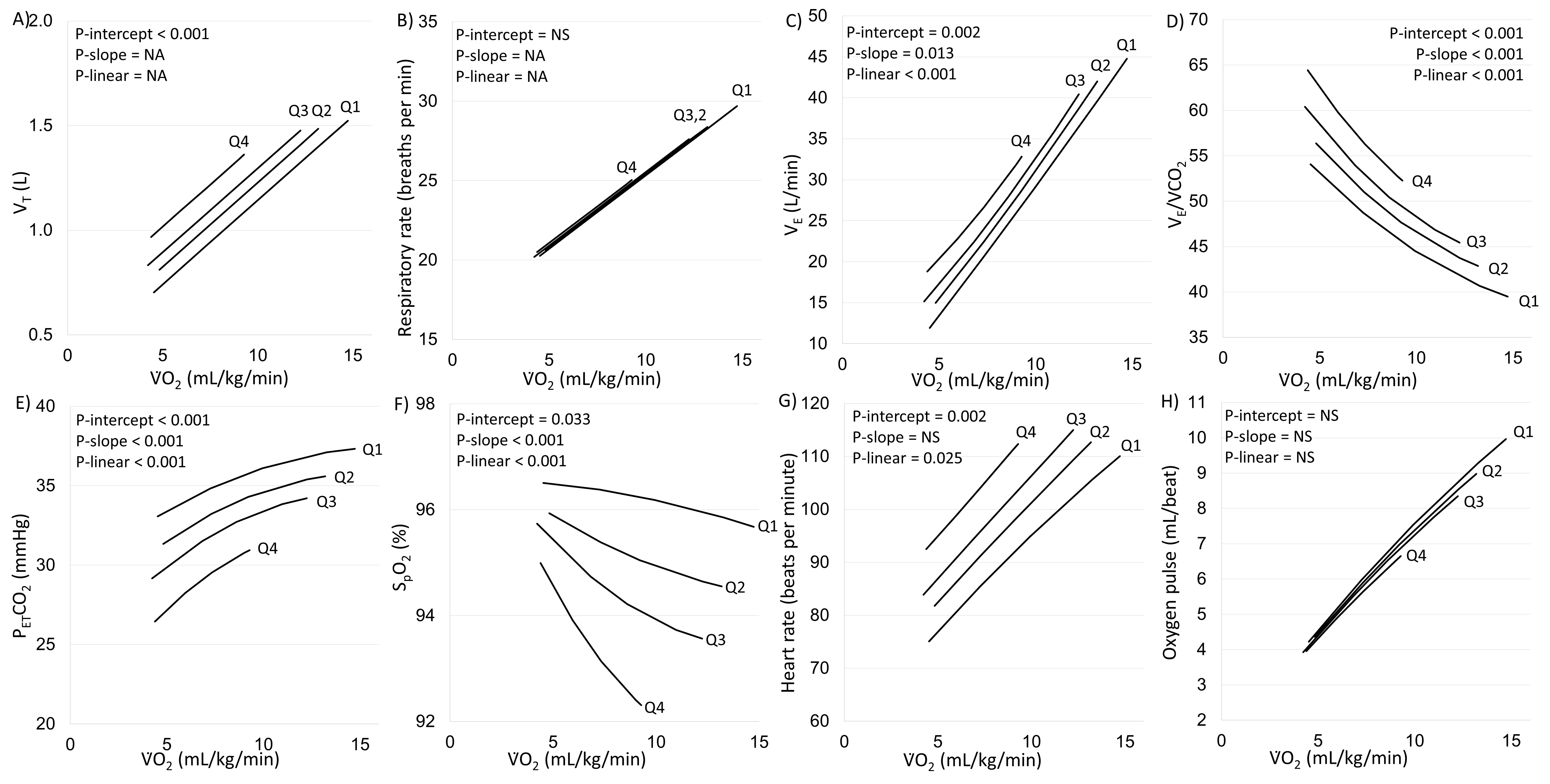


Each panel shows the relationship between a cardiorespiratory response (Y-axis) throughout exercise (X-axis) and percent emphysema (depicted by quartile: Q1: 3.1%; Q2: 8.4%; Q3: 14.5%; Q4: 27.5%). Curves were derived from mixed model regression adjusted for age, gender, BMI, depth of inspiration at CT, smoking status, and pulmonary artery-to-aorta diameter ratio. P-intercept is the probability that percent emphysema predicts no difference in cardiorespiratory response at the intercept. P-slope is the probability that percent emphysema predicts no difference in slope between exercise intensity and cardiorespiratory response. P-linear is the probability that the percent emphysema association with the cardiorespiratory response is linear. NA denotes the model did not require a slope or nonlinear term for optimum fit (See Methods for details). Abbreviations: V_T_ = tidal volume; V̇O_2_ = rate of O_2_ uptake; V̇_E_ = minute ventilation; V̇CO_2_ = rate of CO_2_ output; P_ET_CO_2_ = end-tidal partial pressure of CO_2_; S_p_O_2_ = pulse-oximeter estimated arterial oxy-hemoglobin saturation; CT = computed tomography; and NA = not applicable; and BMI = body mass index.

**e-Table 1.** Percent emphysema and exercise responses at the V̇_E_/ V̇CO_2_ nadir of symptom-limited incremental exercise.

|  | Mean difference at nadir V̇_E_/ V̇CO_2_ in exercise response  per 10% increment in percent emphysema  (95% CI) | | | | |
| --- | --- | --- | --- | --- | --- |
|  | Unadjusted | Model 1 | Model 1 +  GOLD 1-4 | Model 1 +  FEV1 (continuous) | Model 1 +  GOLD A-D |
| Power output – W | **-9**  **(-15 to -3)**  **P=0.003** | **-12**  **(-17 to -6)**  **P<0.001** | **-10**  **(-17 to -3)**  **P=0.004** | **-13**  **(-19 to -6)**  **P<0.001** | **-9**  **(-15 to -3)**  **P=0.002** |
| V̇O_2_ – mL/kg/min | **-1.7**  **(-2.6 to -0.8)**  **p<0.001** | **-2.2**  **(-3.0 to -1.4)**  **P<0.001** | **-1.5**  **(-2.5 to -0.5)**  **P=0.003** | **-1.3**  **(-2.3 to -0.4)**  **P=0.006** | **-1.8**  **(-2.8 to -0.8)**  **P<0.001** |
| V̇_E_/ V̇CO_2_ | **3.1**  **(0.7 to 5.4)**  **P=0.010** | **4.6**  **(1.8 to 7.3)**  **P=0.001** | **5.2**  **(2.2 to 8.2)**  **P<0.001** | **4.7**  **(1.0 to 8.3)**  **P=0.012** | **4.0**  **(1.5 to 6.5)**  **P=0.002** |
| P_ET_CO_2_ – mmHg | -1.0  (-2.2 to 0.3)  P=0.120 | **-1.7**  **(-3.3 to -0.2)**  **P=0.028** | **-3.5**  **(-5.2 to -1.8)**  **P<0.001** | **-2.6**  **(-4.4 to -0.7)**  **P=0.007** | **-1.7**  **(-3.2 to -0.1)**  **P=0.035** |

Mean differences in exercise responses at the V̇E/ V̇CO2 nadir estimated by linear regression. Model 1 adjusts for age, gender, BMI, depth of inspiration at CT, and smoking status. GOLD 1-4 is defined by percent predicted FEV_1_, and GOLD A-D defined by symptoms and exacerbation risk (see Methods for details).

Abbreviations: CI = confidence interval; V̇O_2_ = rate of oxygen uptake; V̇_E_ = minute ventilation; CT = computed tomography; V̇CO_2_ = rate of carbon dioxide output; FEV_1_ = forced expired volume in one second; GOLD = Global Initiative for Chronic Obstructive Lung Disease; and BMI = body mass index.
